# Public Libraries As Partners for Health

**DOI:** 10.5888/pcd15.170392

**Published:** 2018-05-24

**Authors:** Eliza D. Whiteman, Roxanne Dupuis, Anna U. Morgan, Bernadette D’Alonzo, Caleb Epstein, Heather Klusaritz, Carolyn C. Cannuscio

**Affiliations:** 1 School of Design, University of Pennsylvania, Philadelphia, Pennsylvania; 2 Center for Public Health Initiatives, University of Pennsylvania, Philadelphia, Pennsylvania; 3 Division of General Internal Medicine, Harbor–UCLA Medical Center, Torrance, California; 4 Department of Family Medicine and Community Health, Perelman School of Medicine at the University of Pennsylvania, Philadelphia, Pennsylvania

## Abstract

**Introduction:**

Public libraries are free and accessible to all and are centers of community engagement and education, making them logical choices as partners for improving population health. Library staff members routinely assist patrons with unmet health and social needs.

**Methods:**

We used a 100-question, self-administered web survey sent to all library directors listed in the Pennsylvania Library Association database (N = 621), to investigate staff interactions with library patrons to address social determinants of health. We conducted statistical comparisons of quantitative responses and a content analysis of open-ended responses.

**Results:**

Respondents (N = 262) reported frequently interacting with patrons around health and social concerns — well beyond those related to literacy and education — including help with employment (94%), nutrition (70%), exercise (66%), and social welfare benefits (51%). Acute emergencies were not uncommon in Pennsylvania’s public libraries, with nearly 12% of respondents having witnessed a drug overdose at the library in the past year. Most respondents felt that their professional training left them inadequately prepared to assist patrons with health and social issues. Although at least 40% of respondents offered some health programming at their library branch, their offerings did not meet the high level of need reflected in common patron inquiries.

**Conclusion:**

The challenges library staff members experience in meeting their patrons’ information needs suggest opportunities for public libraries to advance population health. Library staff members need additional training and resources and collaboration with public health and health care institutions to respond to community needs through effective, evidence-based public health programming.

## Introduction

As centers for community engagement and education, public libraries provide ideal spaces for the transfer of health information, making them logical choices as partners for improving population health. More than 9,000 public library systems across the country (1) host 1.5 billion in-person visits annually (2), exceeding the number of physician office visits by over 50% (3). During those visits, 42% of patrons report using libraries’ digital resources to search for health information (4).

Literacy, a core mission of libraries, is a cornerstone of lifelong health (5,6). Higher literacy, including health literacy, is associated with increased levels of fulltime employment, on-time high school graduation rates, and a twofold reduced risk of uncontrolled diabetes (7–9). Recent research conducted in Philadelphia showed that public libraries often engage in health-related roles that extend beyond circulation of reading materials (10–12). More than a third of inquiries to public librarians include questions about health (13).

Public libraries also serve as places of refuge for vulnerable populations, including people experiencing mental illness, homelessness, immigration challenges, and trauma (12,14,15). Library staff members regularly assist patrons who have unmet health and social needs, but feel ill-equipped to address these patron needs (12). Previous research has focused on librarians’ role in providing disease-specific consumer health information (16); however, little is known from surveys about the extent to which librarians are called on to assist patrons with social determinants of health, such as housing, employment, and education (17).

The objective of our study was to investigate the frequency and methods library staff members use and are familiar with to address the social determinants of health. Our research — one of several steps taken by the Healthy Library Initiative (18) to establish the feasibility of partnering with public libraries to improve population health — can be extended nationally to inform future partnerships between public libraries and the public health sector.

## Methods

Our 100-question web survey used both open-ended and closed-ended queries to assess the nature and frequency of library staff members’ work related to social determinants of health in 6 domains: 1) education and literacy; 2) wellness and mental health (eg, mental illness, drug abuse, sexual identity); 3) social issues (eg, finding food, applying for social benefits); 4) finances, legal aid, and employment (eg, tax preparation, immigration, post-incarceration); 5) health care (eg, health insurance, finding a provider); and 6) housing and domestic issues (eg, homelessness, intimate partner violence). Questions also evaluated staff members’ professional training to assist patrons in those 6 domains. Additionally, the survey included questions about respondents’ job strain, the most rewarding and challenging aspects of their job, and their experiences managing patrons’ acute health and social issues in the library setting (eg, drug overdose, harassment). Survey development was informed by interviews with library patrons and staff members (12). Each member of the study team tested the survey multiple times before launch. Survey completion took approximately 10 to 15 minutes.

Survey invitations were emailed to 621 public library directors in Pennsylvania in a database compiled by the Pennsylvania Library Association. The survey was self-administered by respondents by using REDCap version R8.2.1 (Research Electronic Data Capture), a secure online survey tool hosted by the University of Pennsylvania (19). Respondents were sent weekly reminder emails over the 4-week period during which the survey was open (January–February 2017). Respondents who opened the survey (n = 313) were automatically entered in a drawing to win one of ten $10 gift cards, while those who completed the entire survey (n = 262) were entered to win one of three $100 gift cards.

The invitation to take the survey specified that respondents must have direct contact with patrons. Initial respondents who did not interact directly with patrons (n = 10) were instructed to provide contact information for an appropriate staff member at their library. Our team then sent the new contact an invitation through an individual email link, which limited responses to only one per library.

The survey data were analyzed using Stata version 11.4 (StataCorp LLC) and ArcGIS version 10.3.1(Esri). Descriptive statistics were calculated and one-sample *t* tests of differences between means (significance at *P* < .05) were conducted for closed-ended survey responses, while thematic coding was conducted to analyze open-ended responses. By using library ZIP codes, responses were categorized to assess geographic variability according to the Office of Management and Budget’s metropolitan and nonmetropolitan designation system, which classifies counties as urban or rural based on labor markets (20,21). This study was approved by the University of Pennsylvania Institutional Review Board.

## Results

The overall survey response rate was 50% (313 responses). Of those, 51 were missing ZIP code information and were therefore excluded, leaving a final analytic sample of 262 respondents. Most respondents (79%) worked in public libraries in metropolitan areas (Table 1) (Figure). The mean age of respondents was 51. Respondents were predominantly female (92%), white (93%), and held a master’s degree in library science (60%). All respondents spent face-to-face time with patrons, and some respondents served multiple roles (eg, librarian and director).

**Table 1:** Demographic Characteristics, Respondents (N = 262) to Survey of Pennsylvania Librarians, 2017

Characteristic	N (%)^a^
**Age, y**	
18–39	51 (19.5)
40–55	80 (30.5)
≥56	103 (39.3)
Not reported	28 (10.7)
**Sex**	
Male	19 (7.3)
Female	242 (92.4)
Not reported	1 (0.4)
**Race/ethnicity**	
American Indian and Alaskan Native	1 (0.4)
Asian	1 (0.4)
Black or African American	6 (2.3)
Hispanic	3 (1.1)
White	244 (93.1)
Other	2 (0.8)
Not reported	8 (3.1)
**Metropolitan–nonmetropolitan classification**	
Metropolitan	206 (78.6)
Nonmetropolitan	56 (21.4)
**Education**	
High school and below	12 (4.6)
Some college/BA	56 (21.4)
Master’s or above	194 (74.0)
**Job title**	
Director	178 (67.9)
Manager	28 (10.7)
Librarian	22 (8.4)
Supervisor	19 (7.3)
Other	14 (5.3)
Not reported	1 (0.4)

a Percentages do not total 100% because participants could select more than one answer.

**Figure:**
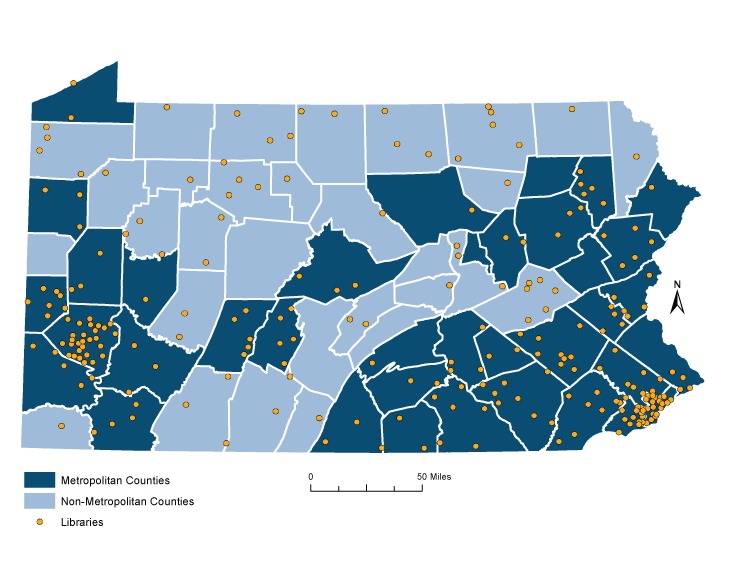
Pennsylvania public library survey respondents (N = 262) by metropolitan and nonmetropolitan area. Data sources include the Economic Research Service (https://www.ers.usda.gov/topics/rural-economy-population/rural-classifications/data-for-rural-analysis/), US Census, Pennsylvania Spatial Data Access (http://www.pasda.psu.edu/), and US ZIP Code Database.

Most respondents reported interacting frequently with patrons in the last month about literacy. Ninety-nine percent said they regularly received inquiries from patrons about computer literacy, 88% about children’s literacy, and 70% about adult literacy. Respondents also reported frequently interacting with patrons on health and social concerns beyond those related to literacy and education, including employment (94%), nutrition (70%), exercise (66%), and social welfare benefits (51%) (Table 2). Both metropolitan and nonmetropolitan respondents reported a similar range of patron queries; few significant geographical differences were observed.

**Table 2:** Pennsylvania Public Libraries That Commonly Address Social Determinants of Health, Respondents (N = 262) to Survey of Pennsylvania Librarians, 2017

Help Offered in Past Month (Percentage Range)	Type of Help Provided	%
≥60	Employment (ie, job searching, resume building, job skills)	94.0
	Financial assistance, such as tax preparation	80.0
	Nutrition	70.0^a^
	Assistance for elderly	66.0
	Exercise	66.0
	Voter registration	63.0^a^
50–59	Transportation	52.0
	Legal aid	51.5
	Assistance enrolling in social benefits and making ends meet	51.0
	Mental illness	50.0
35–49	Drug abuse	48.5
	Physical disabilities	45.0
	Help getting food	40.0
	Safe, affordable housing	39.0
	Homelessness	38.5
25–34	Insurance enrollment	30.5
	Finding a health care provider	28.0^a^
	Assistance after incarceration	25.0
15–24	Contraception	23.0
	Domestic or intimate partner violence	22.0
	Childhood trauma	22.0
	Immigration	18.0^a^
	Sexual and gender identity	18.0

a Higher frequency reported by library staff members in metropolitan areas than in nonmetropolitan areas using Fisher’s Exact Test (*P* < .05).

Among library staff members who participated in this survey, 12% reported having witnessed a patron overdose on drugs in the library in the past year; all worked in libraries in metropolitan areas. Library staff members also reported in open-ended questions that they had encountered other medical emergencies among patrons, including acute mental health crises (eg, panic attacks, self-destructive behaviors), seizures, and strokes.

Respondents were asked to rate how well they felt their professional training had prepared them to address each of the 6 social determinants of health domains. Respondents felt most prepared to address issues related to education and literacy, and felt significantly less prepared to address health and social questions from patrons, such as queries regarding housing, food, and post-incarceration assistance (*t* tests, *P* = <.001) (Table 3).

**Table 3:** Self-Reported Preparedness for Assisting A Patron With Concerns About Various Topics, Respondents (N = 262) to Survey of Pennsylvania Librarians, 2017

Topic Area	Mean Self-Reported Preparedness^a^	*P* Value^b^
Education and literacy	68.8	Reference
Finances, legal aid, employment	54.4	<.001
Wellness and mental health	54.3	<.001
Social issues	52.3	<.001
Health care	48.1	<.001
Housing and domestic issues	46.4	<.001

a Scores ranged from 0, totally unprepared, to 100, fully prepared.

b Mean is statistically different than mean education and literacy score by using *t* test.

To respond to patrons’ diverse needs and inquiries, library staff members reported requesting help from professionals both inside and outside the library. In the past month, respondents most frequently reported consulting other librarians (69%), law enforcement officers (30%), and social workers (24%) for assistance. They less frequently consulted public health professionals (15%), medical professionals (13%), and lawyers (12%). In an open-ended field, respondents outlined additional resources they used to help answer patrons’ health and social questions, including their elected officials, government agencies, community-based organizations (eg, food banks, neighborhood organizations), and other specialists (eg, tax preparers).

Many of the libraries surveyed offered health-related programming, including health insurance enrollment clinics (37%), immunization and health screening clinics (28%), and cooking classes (26%). Almost all respondents (86%) reported offering social activities (eg, book groups, knitting clubs) at their library. Additional health-related programming, reported by 60 respondents in an open-ended field, included weight loss support, healthy snack and free lunch programs, and social services enrollment assistance (eg, food stamps, Social Security), as well as information sessions, speaker series, and health fairs.

Responses demonstrated a mismatch between patron demand and library supply of health-related programming. For example, although 70% of respondents reported frequent patron questions about nutrition, only 37% of libraries reported offering nutrition classes. Similarly, just over 11% of respondents said that their branch offered mental health support groups, whereas about 50% said that they had regular interactions with patrons about mental illness.

Besides not offering programming, respondents reported that they often felt unprepared to offer these services, highlighting a source of job strain. More than half of respondents reported sometimes or often not knowing how to answer their patrons’ health questions (54%) or how to solve the social problems their patrons asked them about (60%). Nearly 50% of survey respondents reported experiencing stress from working with patrons, often acute stress. In the past year, 42% of respondents were harassed by a patron, and 24% felt physically unsafe while at work.

Respondents shared the biggest challenges of their work in open-ended responses, which were classified into 2 broad themes. First, respondents discussed their role as being akin to that of a social worker. Several mentioned the lack of preparation their education had provided for work in a public library, saying, for example, “library science does not prepare one to be a social worker” and “my college training did not provide guidance in working with those with socioeconomic needs. I learned most of my skills 'on the job', through workshops, and working with my friends in social work and law enforcement.” Respondents commonly echoed the sentiment that they struggle with “knowing which social agencies to refer patrons to.”

Second, respondents expressed frustration with resource constraints, stating, for example, “just about all of our problems can be traced back to a lack of sufficient funding.” Respondents offered other examples, such as “not having enough time, staff [members], or resources to do the things for our patrons that we would like to do.” They used creative solutions to overcome staffing constraints by relying on volunteers. This lack of resources is stressful, as respondents described having to “prove their worth” to their communities to maintain funding. Respondents felt that decision makers sometimes mistakenly view libraries simply as a source of free books, rather than recognizing that libraries provide a broad range of services to people who would otherwise fall through the cracks. Respondents described a continuous balancing act to meet multiple patron demands with minimal resources.

For many respondents, the difficulties in working with the public were counterbalanced by an overwhelming sense of reward, largely achieved through meaningful personal interactions and the perception of truly making a difference in people’s lives. Respondents were proud to be associated with a highly respected and valued community organization — one that offers its services free of charge. One respondent said, “I love when someone can finally see progress, whether it is a child just learning to read, a teenager finding an author that makes them love a book, an adult getting and sending their first email. I love that something so simple can impact someone’s life so profoundly.” Another wrote, “[W]e are making a positive impact on patrons’ lives throughout the community by providing a safe, warm environment and numerous opportunities to socialize . . . ” Finally, one respondent stated, “I truly believe in the power of libraries and the positive impact they have on people’s lives. It is great to feel good about the work you do — it can help sustain you in difficult times.”

## Discussion

This survey, the first of its kind in a statewide sample, documents and reinforces the evidence that public libraries address the social determinants of health. The survey was distributed to all public library directors and had a high response rate across both metropolitan and nonmetropolitan areas, suggesting generalizability across Pennsylvania, the fifth most populous state in the United States. To our knowledge, ours is also the first study to quantify the frequency of drug overdose in public libraries, which were reported by more than one in 10 survey respondents as occurring in the past year. As highlighted in the press (22), libraries are facing fallout from the country’s opioid crisis. Some libraries, including branches in the Free Library of Philadelphia, have responded to this epidemic by stocking naloxone and training staff members to use it, while other libraries have limited patron bathroom time to deter use of libraries as drug injection sites. Because libraries are free and open to all, they host some of the most vulnerable people, including active drug users. This survey highlights the need to develop policies, systems, and support for public libraries and their staff members who face acute emergencies and the chronic burden of addiction in their communities.

In addition, public libraries consistently address the social determinants of health. Previous studies, largely from the library sciences, focused on public libraries’ role in disseminating consumer health information and found that library staff members regularly helped patrons with their health information needs (16,23–25). Adding to these findings, this study used a public health perspective to report on the crucial position of library staff members in linking vulnerable populations to the health and social support systems they need.

The high percentage of white respondents suggests that library staff members, particularly directors, may not reflect the demographic makeup of their constituents. This could pose challenges when addressing the needs of a diverse population of library patrons. In Pennsylvania, 82% of the population is white, with considerable variation across counties. For example, in metropolitan Philadelphia County, 41% of the population is white, while in nonmetropolitan Potter County, the white population is more than 98%. Addressing racial disparities in the library sciences training pipeline is essential for improving representativeness among library staff members.

Although prior research has documented a higher frequency of assisting patrons with health information needs among urban libraries than among rural libraries (16), our findings suggested otherwise. In Pennsylvania, public libraries across metropolitan and nonmetropolitan communities reported similar challenges in their work to support the social and health needs of their patrons, despite a lack of formal professional training in this arena. The latter finding supports both our prior research in Philadelphia (12) and other studies reporting that librarians feel that they do not receive adequate training to help patrons with health information needs (16,22). Addressing patron concerns is so much a part of everyday work that several library staff members described themselves as de facto social workers (12,26). This study illustrates the importance of librarians as public health allies and the need to improve professional training of library staff members to address these concerns adequately.

Another important contribution of this study is demonstrating that work-related stress and secondary trauma (ie, stress resulting from hearing about another’s trauma) are common among librarians who interact with vulnerable patron populations (27). Because public libraries are accessible to all, their staff members must address the disparate needs of patrons, including children, families, and people experiencing homelessness or mental illness. Despite these challenges, survey respondents consistently reported that working with the public at a pivotal community institution was the most rewarding aspect of their jobs.

To improve library staff training and address job strain, we created and evaluated a community health specialist training program for library staff members in Philadelphia (11). The training includes a case-based curriculum designed to teach staff members how to address the diverse health and social needs of their patrons and how to debrief about traumatic experiences in the workplace. Graduates reported significantly improved self-efficacy in helping their patrons. Continuing education programs like these are necessary to equip library staff members to address the social determinants of health.

In addition to responding to requests from patrons, libraries also serve as sites of educational health programming. However, among libraries surveyed, patron inquiries generally outstripped programmatic offerings in this domain. This disparity between demand and actual health-related programming offered at public libraries represents a promising, untapped area of need. Our research points to widespread use of libraries by community residents for a range of social, economic, health, and educational purposes. Capitalizing on the library’s role in the community is an opportunity to provide needed health and social services in a location many people already choose to frequent.

This study has several limitations. First, our sample of public libraries in Pennsylvania was compiled through a database of library directors provided by the state library association. This database does not include location information for the libraries, so we were unable to determine the number of metropolitan versus nonmetropolitan libraries within the full sample. In addition, by using the Office of Management and Budget metropolitan/nonmetropolitan classification system — a blunt instrument for classifying urban and rural areas — we may be missing important small-area geographic diversity. This was reflected in several respondent responses to open-ended questions in which they spontaneously indicated that they work at a rural library, despite being from a county classified as metropolitan. Nonmetropolitan areas were slightly underrepresented in the study. Twenty-one percent of responses were from libraries located in nonmetropolitan areas, whereas in the state of Pennsylvania, 45% of counties are nonmetropolitan. However, more libraries are likely located in metropolitan areas than in nonmetropolitan areas. In future work, we would also value insights from library staff members serving in a range of roles. Lastly, this study may be affected by social desirability, selection, and self-report bias. The survey should be replicated nationally to assess how findings vary across the country.

Librarians have many different job responsibilities, including addressing the social determinants of health, for which they feel underprepared by their professional training. This study provides evidence of low literacy around the social determinants of health among library staff members and demonstrates the need for improved training to reduce job strain. Given the frequency of inquiries and emergencies involving vulnerable populations at libraries, this study further supports our findings that libraries are logical partners for improving population health (12). To do this effectively requires systems-level changes. On-site trainings are an important first step in improving the self-efficacy of library staff members in assisting their patrons; however, other possible models include reforming library science degree curricula and creating linkages between health professionals and public libraries to offer support to both staff and patrons. Given the broad reach of libraries in diverse communities across the country, partnerships between public libraries and public health offer a promising path to chronic disease prevention and health promotion.
